# A Bibliometric Analysis of the Top 100 Cited Qualitative Research Papers in Dentistry From Scopus (1998-2024)

**DOI:** 10.7759/cureus.73307

**Published:** 2024-11-08

**Authors:** Gurleen Kaur Anand, Shewta Ramesh, Vineetha Karuveettil, Sandra Sudheer, Rita Riya Justus, Sabarinath D

**Affiliations:** 1 Department of Public Health Dentistry, Amrita School of Dentistry, Amrita Vishwa Vidyapeetham, Kochi, IND

**Keywords:** bibliometrics, citation analysis, dentistry, qualitative study design, research trends

## Abstract

This bibliometric analysis aimed to identify trends among the top 100 most cited qualitative research articles in dentistry. Articles were retrieved from the Scopus database and screened based on predefined inclusion and exclusion criteria. The final set of 100 articles was then analyzed in R Studio (RStudio Team, Boston, MA) to assess performance metrics, including publication and citation trends, citation-publication relationships, and science mapping. Science mapping offers insights into citation analysis, co-citation analysis, bibliographic coupling, co-word analysis, and co-authorship patterns. The top 100 articles had citation counts ranging from 11 to 606, with publication dates spanning from 1998 to 2021, and were coauthored by 351 individuals across 35 different journals. Most papers originated from Europe, followed by North America and Asia. The United Kingdom contributed the most articles (27), and BMC Oral Health was the most frequently cited journal. Among the 351 authors, Hallberg U. was the most cited. The predominant study design was descriptive quantitative. Keyword analysis revealed diverse thematic groupings, reflecting the broad scope of qualitative research in dentistry. Key trending topics included dental care, attitude to health, child, health behavior, dental caries, clinical competence, psychological aspects, and motivation. This bibliometric evaluation provides an overview of qualitative research trends in dentistry and offers valuable insights to guide future research, emphasizing the need for high-quality qualitative studies across various dental fields to strengthen evidence-based practice.

## Introduction and background

Research is a systematic pursuit of knowledge aimed at uncovering truths and expanding understanding across various fields. At its core, research is an effort to uncover the truth [1,2]. The three main branches of research methodology are quantitative, qualitative, and mixed methods; each contributes distinct insights to scientific inquiry [3].

Evidence-based healthcare (EBH), an approach in which healthcare professionals rely on the best available scientific evidence to guide patient care, has traditionally prioritized quantitative research to answer questions of effectiveness and association, with synthesized findings shaping clinical practice [4,5]. However, qualitative research is increasingly recognized for its ability to explore the complex, subjective experiences of patients and practitioners, dimensions that significantly influence patient outcomes but are often difficult to quantify. This evolving appreciation positions qualitative research as a complementary and integral component of EBH, enriching our understanding of factors that affect patient behavior, satisfaction, and treatment outcomes [6].

The term "qualitative research" refers to a variety of methods and approaches that are used to gain a deeper understanding of people's experiences [7]. Its purpose is to better understand both "how" and "why" things behind behaviors and interactions, providing unique insights into aspects of care that quantitative data may overlook. In dentistry, for instance, qualitative studies explore patient anxieties, attitudes, and experiences, as well as dental professionals' perspectives on treatment modalities [8,9].

Despite these benefits, an analysis of leading Oral and Maxillofacial Surgery journals from Europe and North America in 2019 found a predominance of quantitative studies focused on surgical treatment efficacy and patient outcomes. However, this focus often neglects the patient’s perspective, leaving aspects such as patient preparedness, perceptions, satisfaction, coping abilities, and overall wellness underexplored [10]. While qualitative research has gained traction in the dental field, especially in dental public health [11], its use in dentistry is still underrepresented, and many dental researchers have limited exposure to qualitative methodologies [12]. Qualitative insights not only advance patient-centered care but have also successfully informed policy changes in oral health care delivery, such as preferences for dental insurance policies in Iran [13,14].

Given the rising but still underdeveloped recognition of qualitative research in health, a comprehensive examination of its current state in the context of dentistry is necessary. This study addresses this need by conducting a bibliometric analysis of the top 100 most cited qualitative research articles in dentistry. Through bibliometric analysis, which quantitatively evaluates scientific output, this study provides a snapshot of current trends, impact, and evolution in qualitative dental research, guiding future research priorities and methodologies.

## Review

Materials and methods

The top 100 papers using qualitative research methods in dentistry published were selected by querying the scientific database Scopus. The study period was set from 1998 to 2024. The search terms mainly included "qualitative research method," "oral health," and "dentistry" (Table 1).

**Table 1:** Search strategy in the Scopus database

Query (Database: Scopus, Search date: 31-08-2024)	No of hits
((TITLE-ABS-KEY(qualitative research) OR TITLE-ABS-KEY(phenomenology) OR TITLE-ABS-KEY(ethnography) OR TITLE-ABS-KEY(grounded theory) OR TITLE-ABS-KEY(case study) OR TITLE-ABS-KEY(in-depth interview) OR TITLE-ABS-KEY(focus group discussion))) AND ((TITLE-ABS-KEY(dentistry) OR TITLE-ABS-KEY(oral health) OR TITLE-ABS-KEY(dental) OR TITLE-ABS-KEY(oral cavity) OR TITLE-ABS-KEY(teeth))) AND ( LIMIT-TO ( DOCTYPE,"ar" ) ) AND ( LIMIT-TO ( SUBJAREA,"DENT" ) ) AND ( LIMIT-TO ( SRCTYPE,"j" ) ) AND ( LIMIT-TO ( LANGUAGE,"English" ) )	27,562

The inclusion criteria for this bibliometric analysis were original research articles in the field of dentistry that utilized qualitative research methods as the primary approach. Eligible study designs included, but were not limited to descriptive qualitative research, ethnography, grounded theory, and phenomenology. The selected studies covered a wide range of topics within dentistry, including patient care, dental practice, dental education, and other relevant subfields, allowing for a comprehensive overview of qualitative research in the discipline. Studies were excluded if they employed mixed-method or quantitative approaches as the primary methodology or if they were review articles, commentaries, or editorials. Studies where qualitative methods were not central to the research focus were also excluded to ensure consistency with the goals of this bibliometric analysis.

The selected articles were exported into a saved list in Scopus, where they were ranked by citation count in descending order, resulting in the selection of the top 100 articles. The credibility and impact of each publication were assessed based on their citation counts.

Bibliometric analysis was performed using the Bibliometrix package in R Studio (version 4.2.3; RStudio Team, Boston, MA). The analysis focused on two main areas: performance analysis and science mapping. Performance analysis provided metrics related to publications, citations, and citation-publication relationships. Science mapping included citation analysis, co-citation analysis, bibliographic coupling, co-word analysis, and co-authorship networks.

Two independent reviewers (GK, SR) conducted the screening of the selected articles according to the predefined inclusion and exclusion criteria. Bibliometrix provided information on article titles, citation counts, geographical distribution, authorship, year of publication, publication trends, keywords, funding sources, and the journal. Collaboration analysis was employed to identify co-authorships and map the collaboration networks among authors, institutions, or countries. The study designs are categorized into descriptive qualitative study, phenomenological study, ethnographic study, case study, and grounded theory. The selected data was imported into the Bibliometrix of the R Studio, and the findings were imported and reported. The study was reported using the BIBLIO checklist, specifically designed for bibliometric analysis [15].

Results

The total citation counts of the selected 100 articles ranged from 11 to 606. Between 1998 and 2021, the top 100 most cited articles were published. The analysis indicated an annual growth rate of 1.78% in the number of publications, and the average number of citations per document was 46.98. Of the 100 cited publications, 96 were co-authored publications, and the sole-authored publications were four in number (Table 2).

**Table 2:** Characteristics of 100 top-cited articles

Main information about the data	
Timespan	1998-2021
Sources	35
Documents	100
Annual growth rate %	1.78
Document average age	12.8
Average citations per doc	46.98
References	3516
Document contents	
Keywords Plus (ID)	767
Author’s keywords (DE)	277
Author information	
Authors	351
Authors of single-authored docs	4
Authors collaboration	
Single-authored docs	4
Co-Authors per doc	4.05
International co-authorships %	18
Document types	
Article	100
Review	0

Productivity per active years of publication was reported to be 4.76. The selected articles received a total of 4,669 citations, which included 50 self-citations (Table 3).

**Table 3:** Performance analysis of the top 100 most cited articles

Total publications (TP)	100
Number of contributing authors (NCA)	351
Sole authored publications (SA)	4
Co-authored publications (CA)	96
Number of active years of publications (NAY)	21
Productivity per active year of publication (PAY)	100/NAY=4.76
Total citations (TC)	466998
Average citations	46.6998
Collaboration index (CI)	3.5
Collaboration coefficient (CC)	0.96
Number of cited publications (NCP)	100
Proportion of cited publications (PCP)	1
Citations per cited publications (CCP)	46.6998
h-index(h)	37
g-index(g)	63
i-index(i)	100

**Figure 1:**
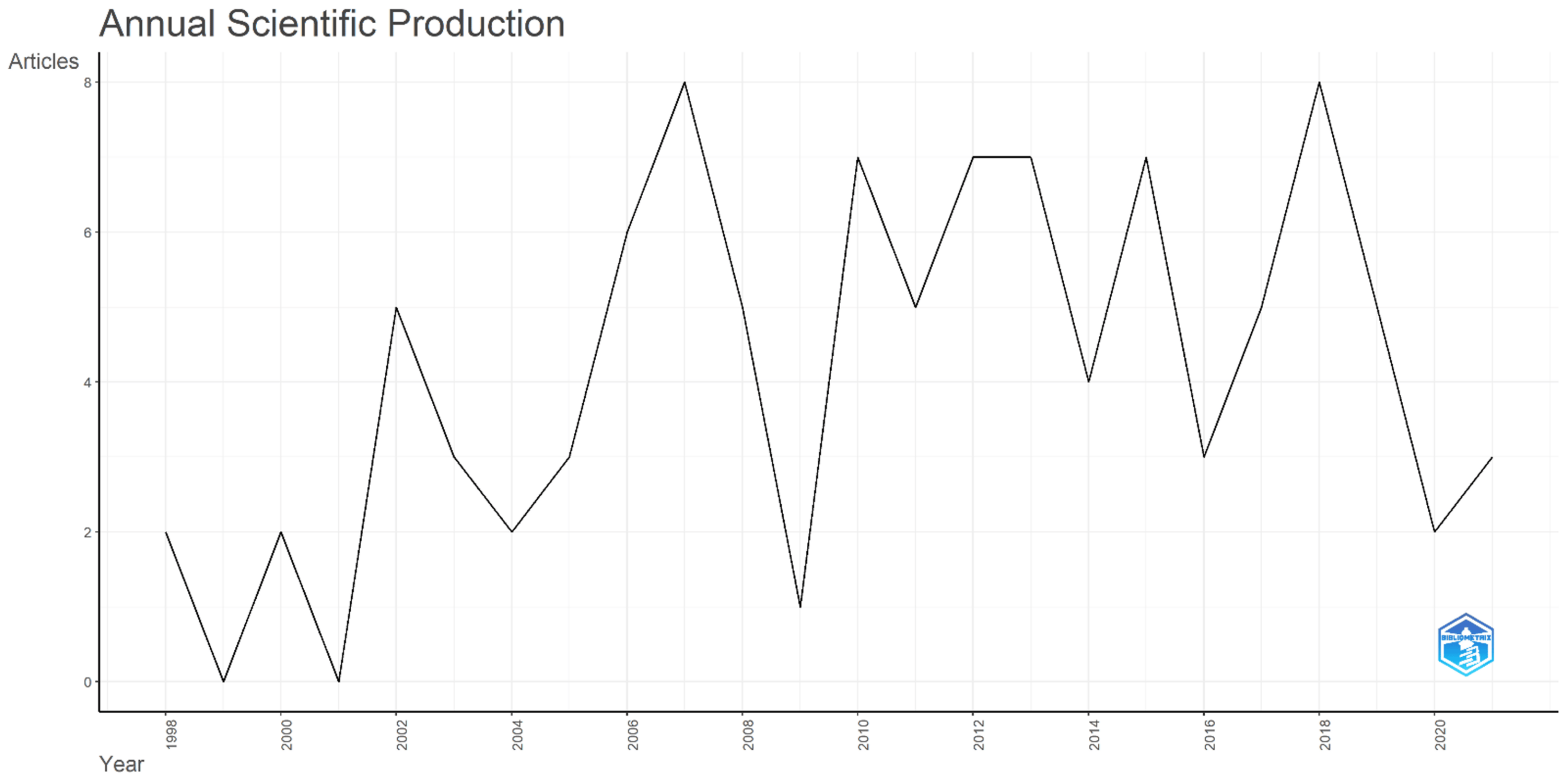
Annual scientific production

The highest mean total citations per year (MTCP) occurred in 1998, with a value of 15.69. The highest cited articles were "Understanding the Culture of Prescribing: Qualitative Study of General Practitioners" and "Patients' Perceptions of Antibiotics for Sore Throats” by Butler CC in 1998 and the article received 606 citations. The oldest paper was published in 1998, and the most recent paper was published in 2021. The highest number of articles (eight) were published in both 2007 and 2018 (Figure 1).

These 100 articles were distributed across 35 different journals. BMC Oral Health emerged as the leading journal, contributing 12 articles with a total of 356 citations, followed by the Journal of Dental Education (11 documents, 59 citations) and British Dental Journal (nine documents, 456 citations). The h-index, g-index, and i-index were found to be 37, 63, and 100, respectively (Table 3).

Hallberg U emerged as the most cited author, with five articles accumulating 112 total citations, followed by Berggren U (four articles, 240 total citations) and Hallberg LRM (four articles, 187 total citations). Hallberg U also demonstrated a high author impact, reflected by an h-index of 18. The most relevant affiliations contributing to the field reported being the University of Sydney nine articles). Most papers were from the United Kingdom (27) and were published in the British Dental Journal and Journal of Dentistry. The National Health and Medical Research Council, the Australian Dental Research Foundation, and the National Institute of Dental and Craniofacial Research were the major funding sources for the studies. The United Kingdom (UK), the United States of America (USA), and Sweden were the top contributors in terms of citations, with totals of 1661, 723, and 624 citations, respectively (Table 4).

**Table 4:** Top 5 countries with the most cited scientific papers

Sr. no	Country	Total citation	Average article citations
1.	United Kingdom	1661	72.2
2.	USA	723	51.6
3.	Sweden	624	32.8
4.	Australia	444	55.5
5.	Canada	208	52

**Figure 2:**
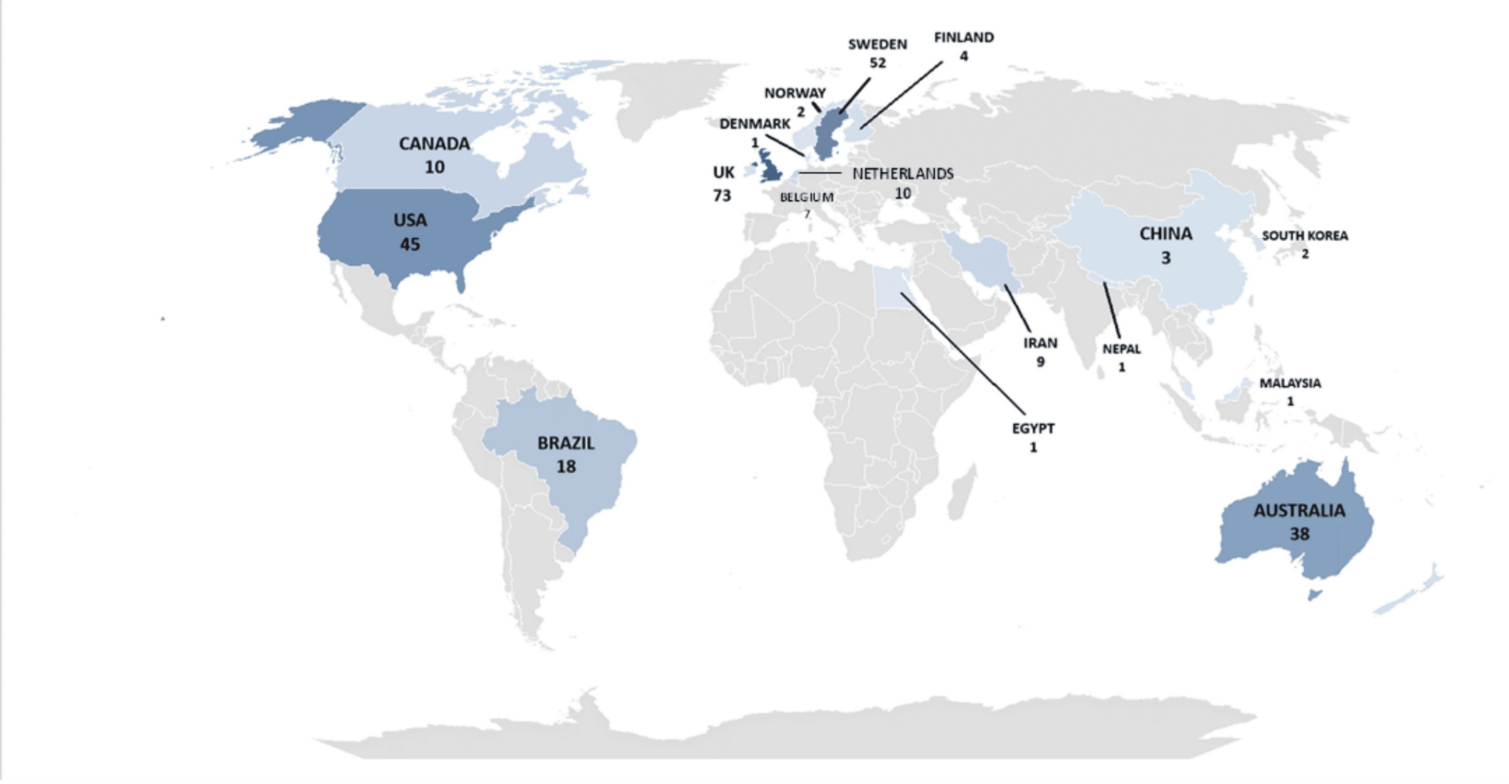
Global distribution of the top 100 cited articles on qualitative research papers used in dentistry

The data indicated that the UK had the highest scientific production (73 publications), followed by Sweden (52) and the USA (45 (Figure 2).

At the continental level, the majority of papers originated from Europe, followed by North America and Asia. Australia, South America, and Africa were represented by one paper each. Descriptive qualitative study design using in-depth interviews or focus group discussion was the most commonly used study design in the 100 articles. The study populations included children, dental students, dentists, the general population, and the elderly.

**Figure 3:**
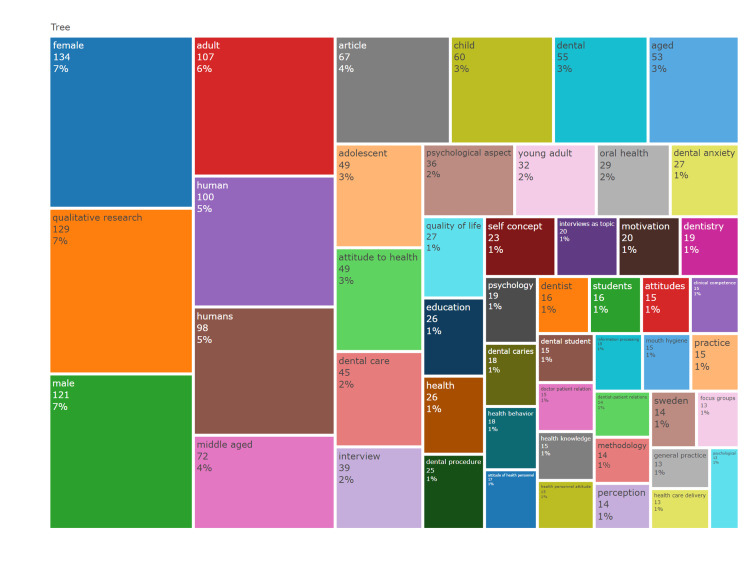
Co-word analysis derived from keywords in the top 100 most cited articles on qualitative research papers used in dentistry

The most frequently occurring keywords included "human" (198 occurrences), "female" (134), "qualitative research" (129), "male" (121), and "adult" (107). The trending topics were reported to be dental care, attitude to health, child, health behavior, dental caries, clinical competence, psychological aspects, and motivation. Regarding the bibliographic coupling, clusters were obtained from sources by measuring the author’s keywords. Co-word analysis revealed the term "child" with the highest cluster frequency and is depicted as a tree map (Figure 3).

The majority of studies employed a descriptive quantitative design (65), followed by grounded theory (23), phenomenological studies (seven), case studies (four), and one ethnographic study. Thematic content analysis using an interview guide or focus group discussion was the predominant method employed for data analysis. Most qualitative studies focused on understanding patients' experiences and perspectives, with a significant emphasis on children's views and experiences regarding oral health, followed by the perspectives of dental students and practitioners.

The analysis of collaborations revealed significant partnerships, particularly between Australia and the UK, Australia, and the USA and Canada. Australia leads in intellectual collaborations (five) with other countries, followed by the UK, USA, and Sweden.

The data underlying the bibliometric analysis are openly available in the Open Science Framework and can be accessed online (osf.io/p4nwx).

Discussion

This bibliometric analysis aimed to identify and analyze the top 100 most-cited qualitative research publications in dentistry, covering articles published between 1998 and 2021, with peak publications in 2007 and 2018. The total citation counts ranged from 11 to 606, indicating a significant variation in the impact of these publications. The average citations per document were 46.98, with an annual growth rate of 1.78%. These metrics reflect the growing interest and recognition of qualitative research within dentistry over the past few decades. A study comparing the prevalence of qualitative methods in Scopus articles from 1996 to 2019 also suggests that qualitative research increased substantially during this period [16].

The UK emerged as the leading contributor, with the highest total citations of 1661 and the highest number of publications of 73 papers. This trend highlights the leading role of UK-based institutions and researchers in advancing qualitative dental research. At the continent level, the majority of the papers were from Europe, followed by North America and Asia. Among African countries, only Egypt contributed to the research field, while Brazil was the sole contributor from South America. This geographic disparity highlights an opportunity to expand qualitative research efforts in these underrepresented regions, especially to bridge gaps in patient-centered care perspectives across diverse populations.

The stark contrast in research funding between high-income and low-income countries underscores the global inequity in scientific advancement. While Western nations invest substantially in research and development (2-4% of gross domestic product (GDP)) [17], low-income regions, particularly South Asia and Sub-Saharan Africa, face significant barriers to research, allocating minimal resources (0.65-0.69% of GDP) [18]. This funding inequity is reflected in the sources supporting the studies included in our analysis, with major contributions coming from Western countries. Key funders include the National Health and Medical Research Council, the Australian Dental Research Foundation, and the National Institute of Dental and Craniofacial Research. This pattern highlights the need for increased investment and support for qualitative dental research in underrepresented regions to address global health disparities effectively.

Descriptive qualitative study designs were the most common methodology employed across the 100 analyzed articles. Qualitative description (QD) is a term used in qualitative research to describe studies that are descriptive in nature [19]. QD differs from other qualitative methods by aiming for a rich, straightforward description of an experience or event, rather than categorizing it into ethnography, grounded theory, or phenomenology. In QD, researchers stay close to the data, presenting informants' experiences in language similar to their own, rather than engaging in reflective or interpretive analysis with existing theories [20,21].

Most of the highly cited qualitative studies in dentistry prioritized understanding patients’ experiences and perspectives, reflecting an increasing emphasis on patient-centered care. Such insights are essential for tailoring treatments and improving patient satisfaction, and they have informed practical changes in dental settings and policies, particularly for vulnerable groups such as children.

The h-index is an important measure that indicates the quality and quantity of a given author’s publications [22]. The g-index is a measure of impact such that the number of articles is cited an average of g times or g2 times. The i10-index calculates the number of significant publications. In this study, h index 37 represents the number of articles that have received at least 37 citations. The g index 63 represents the number of articles that have received citations of around 63 or 126 (g2) citations [23].

Countries engaged in collaborative research tend to attract more citations, suggesting a strong correlation between international cooperation and research impact [24,25]. The UK, USA., and Sweden, which garnered the highest citation counts, also exhibited substantial international research collaboration, particularly involving Australia, which acted as a key player in international research collaborations in our study. This suggests that international cooperation is a key factor in the success of research and that it is essential for countries to collaborate to maximize their research impact.

BMC Oral Health was the most frequently cited journal in qualitative analysis of dental science. It is followed by the Journal of Dental Education and the British Dental Journal. The prominence of these journals highlights a growing recognition of qualitative methodologies within dental research and reflects the journals’ commitment to covering a comprehensive spectrum of oral health topics, from patient care to underlying science.

The most prevalent keywords across the articles were "human", "female", "qualitative research", "male", and "adult". A concept map was used to visually represent keyword co-occurrences, categorizing them into three clusters. However, the utility of word maps and word clouds for deriving meaningful insights is limited due to the absence of standardized interpretation methods. Consequently, in-depth analysis of these keywords is recommended over mere co-occurrence analysis [26].

This study stands out as the first bibliometric analysis focused specifically on trends in qualitative research within the field of dentistry. By examining citation patterns, it provides a broad overview of how qualitative research is conducted in this field and offers insight into its growing recognition. Since citation metrics indirectly reflect a study’s impact, our analysis adds value by highlighting influential studies and foundational topics, which can guide future researchers. Additionally, the findings offer a foundation for subsequent systematic reviews by identifying focal themes and methods, helping researchers select areas that may benefit from a more targeted, comprehensive review.

However, the study has some limitations. The analysis was limited to a single database, Scopus, due to the lack of access to other commonly used bibliometric sources such as Web of Science. Although Scopus offers extensive coverage, including multiple databases could have provided a more complete picture. The selection of only the top 100 most-cited articles - necessary due to the vast literature and resource constraints - also limits the comprehensiveness of the analysis, as less-cited but potentially impactful articles may have been excluded. Furthermore, this study did not perform a critical appraisal to evaluate the methodological quality of each article, given that the objective was to identify trends rather than to inform practice recommendations or policy changes directly.

## Conclusions

This bibliometric analysis has successfully identified the top 100 most-cited qualitative research articles in dentistry, highlighting a significant contribution from UK-based authors and the prevalence of BMC Oral Health as a leading journal in this field. These findings underscore the vital role of qualitative research in capturing patient perspectives, ultimately informing evidence-based practices that can lead to improved oral healthcare outcomes. As the field continues to evolve, further qualitative research is essential to deepen our understanding of patient experiences and to tailor oral healthcare practices to meet the diverse needs of various populations.

## Appendices

**Table 5:** The BIBLIO checklist for reporting the bibliometric reviews of the biomedical literature

Section/Topic	Item No.	Checklist item	Reported on page No.
Title			
Identification	1	Identify the report as a bibliometric review in the title.	1
Issues/topics	2	Indicate the key issues/topics under investigation and coverage of time period.	1
Abstract			
Structured summary	3	Structured summary including (as applicable): background, methods, results (key findings) and conclusions.	1
Introduction/ Background			
Justification/ Rationale/ Explanation	4	Present review of existing knowledge and epidemiological information.	2-3
Objectives	5	Statement of the objective (s) or question (s).	3
Methods			
Search engines (data sources)	6	Describe all information sources (such as electronic databases, contact with study authors, trial registers or other grey literature sources).	3
Search strategy	7	Keywords and systematization criteria (date of search, language, type of document) for the search.	3
Time period	8	The period that the review covers and the justification.	
Eligibility criteria	9	Describe all inclusion and exclusion criteria; languages; study design, type of publication and time period.	3
Data refinement (data selection procedure)	10	Remove the irrelevant articles; inspection to eliminate duplicate and unrelated articles (after evaluation of the title, abstract and content).	3
Quality assessment (optional)	11	Assessment of papers by three authors and the use of assessing checklists.	NA
Data synthesis	12	Describe the methods used for summarizing, handling, synthesis, tabulations or schematic displays. Describe how the data were analysed.	3-4
Results			
Descriptive findings (statistics)	13	- Provide details of the search and selection process in a flow diagram. - Number of citations retrieved (number of publications, year of publication, type of documents, country of publication, articles with the highest impact, most impactful authors, most impactful articles, authors with the highest production, top journals, top institutions, …)	4-11
Schematic map and trend	14	Summarize and/or present the schematic maps and trends using appropriate software to present citations, journals, authors, top journals, time trends, emerging literature, and any relevant indicators (as applicable) [1-5].	8-11
Tabulation and summarizing the findings	15	General recommendation: Studies under consideration could be summarized and organized by different subtitles and different scenarios. Regardless, results need to be presented in separate tables covering each subtitle. The following are some options that could help to summarize the findings. Option 1: - Start the presentation with a historical view [when and who first published on the topic]. - Report on review papers. The result should be listed in a separate table. Also, specify the review type (scoping review, narrative review, systematic review, and meta-analysis). - Summarize the findings according to the study designs and main study types. Option 2: - Start the presentation with a historical view [when and who first published on the topic]. - Report on review papers. The result should be listed in a separate table. Also, indicate the review type (scoping review, narrative review, systematic review, and meta-analysis) should be specified. - Summarize the findings according to outcome measures or populations. For example, see [6]. Option 3: - Start the presentation with a historical view [when and who first published on the topic]. - Report on review papers. The result should be listed in a separate table. Also, specify the review type (scoping review, narrative review, systematic review, and meta-analysis). - Summarize the findings according to concept [7]. Option 4. - Start the presentation with a historical view [when and who first published on the topic]. - Report on review papers. The result should be listed in a separate table, and also specify the review type (scoping review, narrative review, systematic review, and meta-analysis). - Summarize the findings according to different subtitles relevant to the main topic [8].	4-11
Synthesis of findings	16	Synthesize the findings as much as possible, find the gap, and propose a model, hypothesis, etc. (if applicable).	NA
Discussion			
Summary of evidence	17	Summarize the main findings. The findings should be presented in more "general" or "accessible" terms.	11
Interpretation	18	Include interpretation consistent with results. Explanations for observed outcomes, similarities, and differences reported would be essential.	11-13
Strengths and limitations	19	Discuss the strengths and limitations.	13-14
Conclusion(s)	20	Provide a general interpretation of the results with respect to the review questions and objectives, as well as potential implications.	14
1. McDougal L, Dehingia N, Cheung WW, Dixit A, Raj A. COVID-19 burden, author affiliation and women's well-being: A bibliometric analysis of COVID-19 related publications including focus on low-and middle-income countries. eClinicalMedicine 2022; 52: 101606. 2. Henstock L, Wong R, Tsuchiya A, Spencer A. Behavioral theories that have influenced the way health state preferences are elicited and interpreted: A bibliometric mapping analysis of the time trade-off method with VOSviewer visualization. Front Health Serv 2022; 2: 848087. 3. Bodea F, Bungau SG, Negru AP, Radu A, Tarce AG, Tit DM, et al. Exploring new therapeutic avenues for ophthalmic disorders: Glaucoma-related molecular docking evaluation and bibliometric analysis for improved management of ocular diseases. Bioengineering 2023; 10(8): 983. 4. Sang XZ, Wang CQ, Chen W, Rong H, Hou LJ. An exhaustive analysis of post-traumatic brain injury dementia using bibliometric methodologies. Front Neurol 2023; 14: 1165059. 5. Ramli MI, Hamzaid NA, Engkasan JP, Usman J. Respiratory muscle training: a bibliometric analysis of 60 years’ multidisciplinary journey. Biomed Eng Online 2023; 22(1): 50. 6. Akosman I, Kumar N, Mortenson R, Lans A, De La Garza Ramos R, Eleswarapu A,et al. Racial differences in perioperative complications, readmissions, and mortalities after elective spine surgery in the United States: A systematic review using AI-assisted bibliometric analysis. Glob Spine J 2023: 21925682231186759. 7. Tavousi M, Mohammadi S, Sadighi J, Zarei F, Kermani RM, Rostami R, Montazeri A. Measuring health literacy: A systematic review and bibliometric analysis of instruments from 1993 to 2021. Plos One 2022; 17(7): e0271524. 8. Montazeri A. Health-related quality of life in breast cancer patients: A bibliographic review of the literature from 1974 to 2007. J Exp Clin Cancer Res 2008; 27: 32.			
